# Resting Metabolic Rate in Women with Endocrine and Osteoporotic Disorders in Relation to Nutritional Status, Diet and 25(OH)D Concentration

**DOI:** 10.3390/ijerph19053118

**Published:** 2022-03-07

**Authors:** Małgorzata Godala, Ewa Sewerynek, Dominik Maślach, Michalina Krzyżak, Ewelina Gaszyńska

**Affiliations:** 1 Department of Nutrition and Epidemiology, Medical University of Lodz, No.7/9 Żeligowskiego St., 90-752 Łódź, Poland; ewelina.gaszynska@umed.lodz.pl; 2 Department of Endocrine Disorders and Bone Metabolism, Medical University of Lodz, No.7/9 Żeligowskiego St., 90-752 Łódź, Poland; ewa.sewerynek@umed.lodz.pl; 3 Department of Public Health, Medical University of Bialystok, No.37 Szpitalna St., 15-295 Bialystok, Poland; dominikm@umb.edu.pl; 4 Department of Hygiene, Epidemiology and Ergonomics, Medical University of Bialystok, No.2c Mickiewicza St., 15-022 Bialystok, Poland; michalina.krzyzak@umb.edu.pl

**Keywords:** resting metabolic rate, 25(OH)D, diet, metabolic disorders, osteoporotic disorders

## Abstract

There are speculations that vitamin D may be an important regulator of the energy metabolism. The aim of this study was to evaluate the influence of serum 25(OH)D concentration and nutritional status on the resting metabolic rate. The study group consisted of 223 women with endocrine and/or osteoporotic disorders. The control group consisted of 108 women, clinically healthy. The total 25(OH)D concentration level was measured with an assay using chemiluminescent immunoassay technology. Indirect calorimetry was applied to assess the resting metabolic rate. The mean resting metabolic rate was significantly lower in the group of women with metabolic disorders than in the control group. A correlation was found between serum 25(OH)D levels in healthy subjects and the resting metabolic rate. Significantly higher resting metabolic rate was found in women with normal serum 25(OH)D levels in comparison to subjects with deficient vitamin D levels. The control group demonstrated a relationship between body fat tissue and fat-free body mass and the resting metabolic rate. Both 25(OH)D concentration and body composition were factors influencing the resting metabolic rate in the group of healthy subjects. More research is needed to clarify the relationship between vitamin D status and metabolic rate in individuals with endocrine and osteoporotic disorders.

## 1. Introduction

The resting metabolic rate, also called resting energy expenditure, is one of components of the total energy demand and it makes up its greater portion. Apart from basic factors that undoubtedly influence the value of the demand, such as fat body mass and fat-free body mass, gender, age, or ethnicity, the variability of resting metabolism in different groups of patients makes it necessary to search for other factors predicting its value. Energy homeostasis is vital for normal functioning of the body. However, in the event of metabolic changes, depleting adaptive mechanisms disturb its balance. In a study, a group of patients with metabolic diseases demonstrated lower values of the basal metabolic rate, which might indicate that there are factors which lower the rate of energy metabolism in these patients and which are not normally taken into account in the assessment of the resting metabolic rate, such as vitamin and mineral deficiencies or hormone imbalances [1,2,3]. A perusal of the resting metabolic rate literature reveals that considerable information on studies of specific population subgroups, e.g., patients with metabolic disorders, are sparse, especially as far as non-specific factors influencing resting metabolic rate are concerned.

Maintenance of the body energy balance is primarily related to dietary energy supply. However, selected nutrients also contribute to the maintenance of this balance by accelerating or slowing down the metabolic rate. Food intake itself induces energy expenditure related to the so-called thermic effect of food, but there are hypotheses denying the effectiveness of this process in individuals with metabolic disorders [4,5,6]. Nutrients, including proteins, fats, carbohydrates, and dietary fibre, are believed to affect energy expenditure despite there being no conclusive evidence for their effect. In addition to basal metabolism, physical activity is an important component of 24-h energy expenditure. Many studies on a possible influence of physical activity on the resting metabolic rate are contradictory. However, a few authors suggest that the modulating effect of physical activity on the energy metabolism rate occurs when it is accompanied by a decrease in body weight [7,8].

In recent years, vitamin D deficiency, which has long been regarded as a risk factor for many diseases and not only a regulator of calcium-phosphate metabolism, has been widely discussed. Results of many studies have confirmed the very frequent occurrence of serum 25(OH)D deficiency in patients with metabolic syndrome, cardiovascular disease, or endocrine and osteoporotic disorders [9,10]. There are also reports on the role of vitamin D in regulating skeletal muscle tone, aerobic energy metabolism, and lipid metabolism [11]. There is also evidence that vitamin D may be an important regulator of the rate of energy metabolism [12,13,14].

## 2. Objectives

The aim of this study was to evaluate the resting metabolic rate in women with metabolic disorders. Moreover, the influence of diet, serum 25(OH)D concentration, nutritional status, and physical activity levels on the energy metabolism was also evaluated.

## 3. Material and Method

### 3.1. Study and Control Group

A total of 331 women took part in the case-control study. The study group consisted of 223 women, aged 19–81 years (the mean age 64.6 ± 12.8 years) with endocrine and/or osteoporotic disorders. The control group consisted of 108 women, aged 26–72 years (the mean age 61.4 ± 11.3 years), clinically healthy and without the above disorders.

In the study group, osteoporosis was found in 110 subjects (49.3%), endocrine disorders (including hypothyroidism, Hashimoto’s inflammation, inactive nodules) were found in 53 women (23.8%), while 60 women (26.9%) were affected by both these disorders. Patients with hypothyroidism and Hashimoto’s disease were compensated with thyroid hormone replacement therapy.

In the study group, 26 women smoked cigarettes (11.7%), 173 subjects (77.6%) had used vitamin D supplementation at a dose of 1000–2000 UI and calcium supplementation at a dose of 500–600 mg per day for at least three months. In the control group, 31 women (28.7%) used vitamin D supplementation at a dose of 1000–2000 UI for at least three months. None of the women smoked.

The study was conducted according to the guidelines of the Declaration of Helsinki, and approved by the Bioethics Committee of the Medical University of Łodz (No. RNN/556/10/KB).

### 3.2. Physical Activity

In the all studied women, the level of physical activity was assessed using the International Physical Activity Questionnaire (IPAQ) [15]. The authors estimated the so called total physical activity expressed in MET—min/week (Metabolic Equivalent of Work), where one MET represents resting energy expenditure assuming oxygen consumption of 3.5 mL/min/kg body weight.

There are three types of effort, namely light (walking), moderate (with slightly increased respiratory rate and slightly accelerated heart rate, such as carrying light objects, cycling at a normal speed, brisk walking), and intensive (with highly increased respiratory rate and accelerated heart rate, such as aerobics, fast cycling). The time spent on each type of activity in the last week is recorded and only efforts lasting at least 10 min are taken into account.

The questionnaire consists of 27 questions divided into 5 groups, each of which contains detailed questions on intensive, moderate, and walking activities undertaken by the subjects in the last week and related to their work, active mobility, housework, leisure, and sport. Moreover, time spent inactively during a week and at the weekend is also analysed in the questionnaire.

The results were classified according to the following criteria:

1. Insufficient physical activity (less than 600 MET—min/week);
2. Sufficient physical activity (between 600 and 1500 MET—min/week);
3. Increased physical activity (1500–3000 MET—min/week, but less than 3 days per week of intense exercise);
4. High physical activity (above 1500 MET—min/week but at least 3 days per week of intense exercise, or at least 3000 MET—min/week).

The respondents completed the questionnaire with the help of a questioner. The questionnaire was thoroughly discussed prior to the commencement of the study, and particular attention was paid to explanation of the terminology used in the questionnaire and the interpretation of the intensity of physical efforts. For each category, the respondents separately identified the number of days and time spent on intensive and moderate activity as well as walking.

### 3.3. Vitamin D

Fasting blood for laboratory testing was collected from the ulnar vein. The obtained blood samples were used to determine the total 25(OH)D concentration level with an assay using the chemiluminescent immunoassay (CLIA) methodology. A serum 25(OH)D concentration of at least 30 ng/mL was considered normal, whereas the level below 30 ng/mL was considered insufficient (deficient concentration) [16].

### 3.4. Metabolic Rate

Indirect calorimetry was applied to assess the resting metabolic rate, using a Cosmed Fitmate Pro apparatus. A minimum 30-min measurement of absorbed oxygen and exhaled carbon dioxide was performed, and the amount of energy expended was calculated on the basis of the energy equivalent for oxygen.

All the studied women abstained from any strenuous physical activity for at least 24 h prior to the test. The measurement was made in the early morning, in a sitting position, in silence, without artificial light sources, and at room temperature.

Furthermore, using the Harris–Benedict formula, basal metabolism was calculated, then total metabolism, taking the subjects’ level of physical activity into account [17].

### 3.5. Anthropometry

All the subjects had their waist measured, WHR was determined by dividing the waist circumference by hip circumference, and BMI (the body mass index) was determined by dividing body weight expressed in kilograms by height in square metres.

The body composition of the subjects was assessed with electrical bioimpedance analysis (BIA) using a Bodystat 1500 MDD apparatus. Fat body mass and fat-free body mass were measured.

### 3.6. Nutrition Assessment

Food intake was assessed with a 24-h questionnaire, collected three times from each subject (from two weekdays and one holiday). The average intake of energy and particular nutrients was assessed using the computer program Diet 5.0 (license number 52/PD/2013) [17].

### 3.7. Statistical Analysis

A statistical analysis was performed using the Statistica v.13 programme. Descriptive statistics with determination of the mean and standard deviation were made. The analysis of compatibility of the variable distribution with the normal distribution was performed with the application of the Shapiro–Wilk test. When the analysed variables appeared to be incompatible with the normal distribution, the authors used the Mann–Whitney test to compare the study and control groups. For ordinal variables or variables with distribution incompatible with the normal distribution and in order to determine the correlation between variables, the authors applied the Spearman’s rank order correlation with determination of the Spearman’s R coefficient and assessment of statistical significance. *p* < 0.05 was considered significant.

## 4. Results

There were no statistical differences in age, weight, waist circumference, BMI, and WHR between the patients and controls, but subjects with metabolic disorders used vitamin D supplementation significantly more often than healthy women. With regards to the level of physical activity, the subjects in both the groups did not differ significantly. Every sixth respondent demonstrated insufficient physical activity. More than half of the respondents demonstrated a sufficient level. Every fifth respondent demonstrated an increased level of physical activity, whereas a high level of physical activity was noted in the smallest number of women. An analysis of body composition showed q significantly higher content of fat tissue and a lower level of fat-free mass in the group of women with metabolic disorders (Table 1).

The mean resting metabolic rate was significantly lower in the group of women with metabolic disorders than in the control group (1332.7 kcal vs. 1557.8 kcal, Z = 4.4953, *p* = 0.0000). However, it did not differ significantly between subjects with endocrine and osteoporotic disorders (1299.3 kcal vs. 1382.2 kcal, Z = 0.7537, *p* = 0.3582). Similar to the resting metabolic rate, the calculated value of basal metabolism was significantly higher in the control group. In contrast, there were no significant differences in the mean values of total metabolism between subjects with metabolic disorders and healthy subjects.

**Figure 1:**
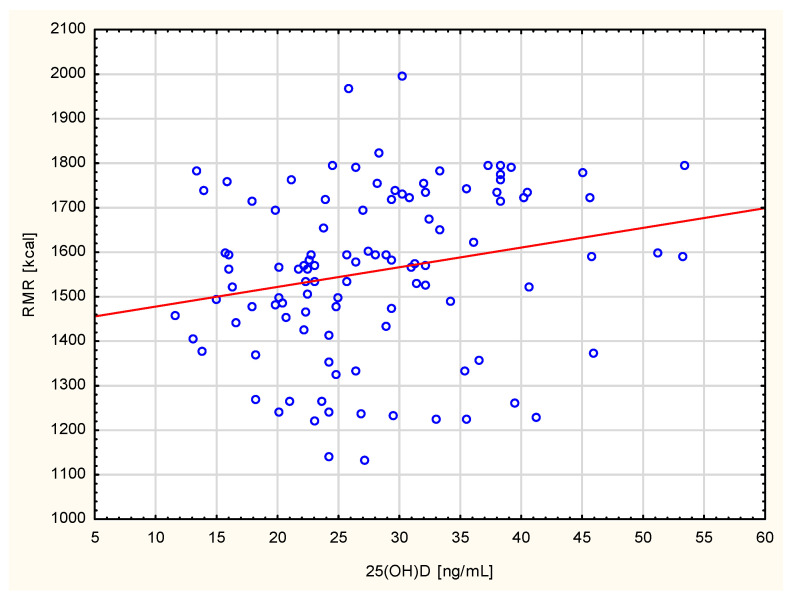
Correlation between 25(OH)D and RMR in healthy women.

A correlation was found between serum 25(OH)D levels in healthy subjects and the resting metabolic rate (R = 0.27, *p* = 0.0000). This relationship was not confirmed in the group of women with metabolic disorders (Figure 1). Moreover, a significantly higher resting metabolic rate was found in women with normal serum 25(OH)D levels in comparison to subjects with deficient vitamin D levels (1538.5 kcal vs. 1284.9 kcal, Z = 3.7352, *p* < 0.0001), regardless of any occurrence of endocrine and osteoporotic disorders (Table 2). Besides, women with a high resting metabolic rate appeared to demonstrate significantly higher 25(OH)D concentration than women with a low and moderate resting metabolic rate (28.2 ± 3.8 ng/mL vs. 20.5 ± 3.1 ng/mL, Z = 5.0276, *p* < 0.0001), regardless of any occurrence of metabolic disorders. The women with a high resting metabolic rate more often demonstrated sufficient 25(OH)D concentration than women with a low and moderate resting metabolic rate (28.2 ± 3.8 vs. 20.5 ± 3.1, Z = 5.0276, <0.0001). They were characterised by a higher intake of proteins and fibre (Table 3).

The authors observed a correlation between the intake of selected nutrients and the resting metabolic rate. A positive correlation was found between the total dietary intake of vitamin D and the resting metabolic rate in healthy subjects (R = 0.25, *p* < 0.0001). This observation was not confirmed in the patient group. In addition, all studied female subjects showed a positive correlation between resting metabolism and the total dietary intake of protein (R = 0.22, *p* < 0.0001), plant protein (R = 0.23, *p* < 0.0001), starch (R = 0.24, *p* = 0.0000), fibre (R = 0.21, *p* < 0.0001), potassium (R = 0.21, *p* < 0.0001), phosphorus (R = 0.23, *p* < 0.0001), and zinc (R = 0.25, *p* < 0.0001). There was also a relationship between the resting metabolic rate and the intake of vitamins, including thiamin (R = 0.21, *p* < 0.0001), riboflavin (R = 0.21, *p* < 0.0001), niacin (R = 0.21, *p* < 0.0001), pyridoxine (R = 0.22, *p* < 0.0001), and folic acid (R = 0.26, *p* < 0.0001).

Physical activity level and body composition also affected the resting metabolic rate. A significantly higher resting metabolic rate was found in subjects with increased and high levels of physical activity compared to the other female subjects (1631.6 kcal vs. 1377.3 kcal, Z = 6.8451, *p* < 0.0001), regardless of an occurrence of metabolic disorders. In contrast, the control group demonstrated a relationship between body fat tissue (R = −0.21, *p* < 0.0001) and fat-free body mass (R = 0.32, *p* < 0.0001) and the resting metabolic rate. The group of women with osteoporotic and endocrine disorders showed a similar relationship only for fat-free body mass (R = 0.28, *p* < 0.0001).

## 5. Discussion

Our study showed a positive relationship between vitamin D concentration and the resting metabolic rate in healthy subjects. This relationship was not confirmed in the group of women with metabolic disorders. Moreover, a significantly higher resting metabolic rate was found in women with normal serum 25(OH)D concentration compared to subjects with deficient serum 25(OH)D concentration, regardless of concomitance of osteoporotic and endocrine disorders. Our results therefore suggest an association between vitamin D nutritional status and the pace of energy metabolism. Studies conducted on mice showed that vitamin D is associated with regulation of energy metabolism rates [18,19]. In contrast, there are hardly any studies verifying the effect of vitamins on human energy expenditure and their results are often contradictory. A study by Marcotorchino et al. did not show a significant effect of vitamin D on energy metabolism. Nevertheless, it should be noted that this was a short-term observation [20]. Montenegro et al. included physically active adults in their study but they did not observe any effect of vitamin D supplementation on resting metabolism, the body composition and skeletal muscle function [11]. In other studies, this relationship was confirmed. Calton et al. demonstrated that serum vitamin D concentration is an independent predictor of the resting metabolic rate in Australian adults [21]. In this study, it was shown that an increase in 25(OH)D concentration from 40 to 75 nmol/L results in a change pertaining to daily resting metabolism by approximately 200 kJ per day, which simultaneously contributes to weight loss. The mechanism of the thermogenic action of vitamin D is not fully understood. There are hypotheses about its direct effect through nVDR or by influencing the skeletal muscle size and function, which results in an increased metabolic rate [18,22]. There is also speculation that vitamin D may affect the mitochondrial function and modulate resting oxygen consumption, which is positively correlated with the resting metabolic rate [23].

The influence of physical exercise on the resting metabolic rate is still a controversial and debated issue. In our study, patients with an increased and high level of physical activity were characterised by a significantly higher resting metabolic rate. A relationship between body composition and resting metabolism was also confirmed. Both in the group of healthy and diseased women, the content fat-free body mass positively correlated with the values of resting metabolism. Results of our study confirmed the observations of some authors but were contradictory to observations made by others. Some studies deny the influence of physical exercise on the resting metabolic rate [14,24]. Other studies confirmed this relationship, both in healthy subjects and those with metabolic disorders [3,25,26]. The body composition of the subjects is an important issue taken into account when assessing an influence of physical activity on the rate of energy metabolism. A study by Karstoft et al. showed that regardless of the type of undertaken physical activity, the body composition did not affect the changed resting metabolic rate. The rate was unchanged if the body composition was unchanged, too. Both fat and fat-free body mass affected the resting metabolic rate. However, when evaluating the effect of physical exercise on energy metabolism, only the changes in fat body mass, induced by physical exercise, determined changes in resting metabolism. A decrease in the volume of the fat body mass was accompanied by a decrease in the value of resting metabolism [7]. However, in our study, fat body mass was inversely related to resting metabolism, but only in the group of healthy women. Among women with metabolic disorders, only fat-free body mass correlated positively with values of the resting metabolic rate. Differences in the opinion on the possible influence of physical activity and nutritional status on the resting metabolic rate have not yet been clarified. Perhaps the type of programmed physical activity, its duration and intensity, as well as its influence on changes in body composition are important in assessing the potential impact on the body energy metabolism. In studies assessing the possible influence of physical activity on resting metabolism, changes in the body mass of the subjects were analysed, suggesting that they are a necessary condition to modify energy metabolism [7,8].

The effect of selected nutrients on the rate of energy metabolism has been discussed for long. In our study, apart from vitamin D, we found a relationship between the resting metabolic rate and the total intake of protein, plant protein, starch, fibre, potassium, phosphorus, zinc, and B vitamins. The effect of a diet, especially of selected nutrients, on the body energy metabolism rate is mainly related to the so-called thermic effect of food, i.e., generation of heat after a meal (non-shivering thermogenesis). It can result in an increase in the total energy expenditure and a decrease in body weight. It depends, among other things, on the quality composition of the meal, its energy value, and the time of its consumption. In professional literature, proteins are most often mentioned as nutrients that influence human energy expenditure, although their influence on resting metabolism has not been clearly confirmed. However, a high protein intake is often associated with a decrease in body weight, which may explain this relationship [4,6]. On the other hand, carbohydrates and fats induce a significantly lower value of non-shivering thermogenesis than proteins [27,28,29]. However, studies showed a relationship between higher fibre intake and an enhanced thermic effect [4], which was also confirmed in the present study. In our study, the total intake of protein and plant protein also correlated with an increase in the resting metabolic rate. It is estimated that the intake of protein at the level of 11–30% of the total energy value of the diet increases the level of non-shivering thermogenesis up to 30%, while a further increase in the protein intake (more than 30% of the energy value of the diet), does not cause a further thermal effect [30]. The source of protein is also significant, due to differences in metabolism of proteins of different origin. Differences in the rate and pace of changes in non-shivering thermogenesis after consumption of different types of proteins remain to be explained. A higher increase in non-shivering thermogenesis after the consumption of whey proteins than casein was shown, whereas differences between the effects of whey and soy proteins were not explained [29].

The present study has some limitations. It would be possible to obtain reliable data on the effect of undertaken physical activity on the resting metabolic rate if this rate were measured shortly after the activity. The authors did not determine how much the undertaken physical activity affected the body composition, which to some extent conditions the rate of energy metabolism and is also related to vitamin D nutritional status.

## 6. Conclusions

The 25(OH)D concentration, intake of selected nutrients, and body composition were factors influencing the resting metabolic rate in the group of healthy subjects assessed here. More research is needed to clarify the relationship between vitamin D status and metabolic rate in individuals with endocrine and osteoporotic disorders.

## Figures and Tables

**Table 1:** Characteristics of the participating women.

Characteristics	Study Group *n* = 223	Control Group *n* = 108	Z, *p* Mann–Whitney Test
	Mean ± SD/*n* (%)	Mean ± SD/*n* (%)	
Age [years]	64.6 ± 12.8	61.4 ± 11.3	4.2306, 0.4673
Body Mass Index (BMI) [kg/m^2^]	27.8 ± 5.5	26.1 ± 5.8	1.1365, 0.7671
Waist Hip Ratio (WHR)	0.8 ± 0.1	1.0 ± 0.1	1.4235, 0.5328
Waist circumference [cm]	88.8 ± 12.9	87.2 ± 7.2	1.1463, 0.5659
Regular vitamin D supplements user [*n* (%)]	173 (77.6)	31 (28.7)	4.5638, <0.0001
Physical activity			
Insufficient	38 (17.1)	17 (15.7)	0.8735, 0.5374
Sufficient	120 (53.8)	55 (50.9)	0.3467, 0.3859
Increased	44 (19.7)	20 (18.5)	0.7436, 0.4783
High	21 (9.4)	16 (14.8)	0.8834, 0.7739
Body composition			
Body mass [kg]	71.5 ± 14.7	73.5 ± 7.9	0.9453, 0.6233
Fat free mass [kg]	46.1 ± 6.2	49.1 ± 5.2	2.9964, <0.0001
Fat mass [kg]	32.4 ± 13.5	28.4 ± 8.3	3.5762, <0.0001
Fat mass [%]	39.7 ± 8.7	36.2 ± 4.7	4.0276, <0.0001
Metabolic Rate			
Resting Metabolic Rate [kcal/d]	1332.7 ± 309.9	1557.8 ± 187.3	7.0590, <0.0001
Basal Metabolic Rate [kcal/d]	1453.2 ± 245.6	1574.0 ± 134.9	5.0123, <0.0001
Total Metabolic Rate [kcal/d]	2409.7 ± 254.7	2427.5 ± 288.7	0.3632, 0.0762
Slow [*n* (%)]	92 (41.3)	12 (11.1)	3.9432, <0.0001
Normal [*n* (%)]	83 (37.2)	14 (13.0)	2.9964, <0.0001
High [*n* (%)]	48 (21.5)	82 (75.9)	4.0275, <0.0001
Dietary intake			
Energy [kcal/day]	1415.7 ± 447.4	2127.2 ± 1182.3	4.2349, <0.0001
Proteins [g/day]	57.9 ± 19.5	99.6 ± 45.8	2.9836, <0.0001
Proteins [% total energy intake]	16.7 ± 4.5	19.4 ± 3.5	2.7906, <0.0001
Carbohydrates [g/day]	189.0 ± 61.0	268.8 ± 139.8	6.4326, <0.0001
Carbohydrates [% total energy intake]	50.3 ± 8.8	48.1 ± 8.8	0.7683, 0.7639
Fats [g/day]	53.4 ± 24.3	79.3 ± 57.7	3.9465, <0.0001
Fats [% total energy intake]	32.6 ± 8.1	32.1 ± 7.2	0.8032, 0.4327
MUFA [g/day]	21.2 ± 10.6	30.8 ± 28.9	5.0348, <0.0001
PUFA [g/day]	8.1 ± 4.2	10.3 ± 7.1	3.9674, <0.0001
SFA [g/day]	20.2 ± 11.3	34.4 ± 28.4	4.4876, <0.0001
Fiber [g/day]	15.7 ± 5.9	24.3 ± 11.2	4.0824, <0.0001
Vitamin D (without supplements) [µg/day]	3.7 ±2.3	7.4 ± 5.6	2.9987, <0.0001
Total vitamin D (including supplements) [µg/day]	38.2 ± 15.0	21.1 ± 13.6	3.9075, 0.0028
Serum concentration			
25(OH)D Total [ng/mL]	25.9 ± 11.8	28.1 ± 9.1	2.1808, 0.0029
25(OH)D deficiency [*n* (%)]	149 (66.8)	68 (62.9)	0.8725, 0.8043

**Table 2:** Characteristics of the participating women according to 25(OH)D concentration.

Characteristics	25(OH)D < 30 ng/mL *n* = 217	25(OH)D ≥ 30 ng/mL *n* = 114	Z, *p* Mann–Whitney Test
	Mean ± SD/*n* (%)	Mean ± SD/*n* (%)	
Age [years]	65.2 ± 10.2	61.9 ± 9.8	3.2904, 0.7325
Body Mass Index (BMI) [kg/m^2^]	26.9 ± 4.9	26.3 ± 2.9	1.1763, 0.7781
Waist Hip Ratio (WHR)	0.8 ± 0.3	0.9 ± 0.9	1.6233, 0.6371
Waist circumference [cm]	87.9 ± 11.2	86.2 ± 6.9	1.1393, 0.4938
Regular vitamin D supplements user [*n* (%)]	94 (43.3)	110 (96.5)	2.5032, <0.0001
Physical activity			
Insufficient	42 (19.4)	13 (11.4)	0.2756, 0.6314
Sufficient	115 (53.0)	60 (52.6)	0.2961, 0.7851
Increased	43 (19.8)	21 (18.4)	0.5826, 0.4764
High	17 (7.8)	20 (17.6)	0.7463, 0.3753
Metabolic Rate			
Resting Metabolic Rate [kcal/d]	1284.9 ± 279.1	1538.5 ± 217.8	3.7352, *p* < 0.0001
Basal Metabolic Rate [kcal/d]	1351.2 ± 179.8	1602.0 ± 171.6	1.7821, <0.0001
Total Metabolic Rate [kcal/d]	25,024.2 ± 174.8	2479.5 ± 308.1	0.3037, 0.1772
Slow [*n* (%)]	89 (41.0)	15 (13.2)	2.7472, <0.0001
Normal [*n* (%)]	86 (39.6)	11 (9.6)	1.9071, <0.0001
High [*n* (%)]	42 (19.4)	88 (77.2)	3.1234, <0.0001
Dietary intake			
Energy [kcal/day]	1493.7 ± 507.8	2221.8 ± 982.4	3.9843, <0.0001
Proteins [g/day]	55.8 ± 15.9	90.6 ± 49.1	1.9037, <0.0001
Proteins [% total energy intake]	15.9 ± 6.1	20.7 ± 3.1	2.2972, <0.0001
Carbohydrates [g/day]	177.9 ± 60.5	251.8 ± 143.1	3.7896, <0.0001
Carbohydrates [% total energy intake]	53.3 ± 6.8	49.7 ± 7.5	0.7323, 0.6739
Fats [g/day]	57.4 ± 21.5	81.3 ± 49.7	1.0467, <0.0001
Fats [% total energy intake]	30.7 ± 9.1	34.1 ± 8.1	0.2092, 0.1357
MUFA [g/day]	20.9 ± 9.9	32.1 ± 25.9	4.1287, <0.0001
PUFA [g/day]	8.7 ± 4.4	12.1 ± 5.7	3.0631, <0.0001
SFA [g/day]	24.2 ± 6.3	37.4 ± 19.7	4.8671, <0.0001
Fiber [g/day]	13.9 ± 6.2	26.3 ± 9.7	3.8021, <0.0001
Vitamin D (without supplements) [µg/day]	3.1 ± 2.9	8.2 ± 4.8	1.9042, <0.0001
Total vitamin D (including supplements) [µg/day]	36.7 ± 16.3	20.3 ± 14.1	3.0472, 0.0017

**Table 3:** Characteristics of the participating women according to the level of metabolic rate.

Characteristics	Slow and Normal Metabolic Rate *n* = 201	High Metabolic Rate *n* = 130	Z, *p* Mann–Whitney Test
	Mean ± SD/*n* (%)	Mean ± SD/*n* (%)	
Age [years]	67.4 ± 11.1	59.9 ± 11.4	1.2307, 0.0025
Body Mass Index (BMI) [kg/m^2^]	28.3 ± 6.2	24.9 ± 4.7	1.1763, 0.0018
Waist Hip Ratio (WHR)	1.1 ± 0.9	0.9 ± 0.7	1.0537, 0.7872
Waist circumference [cm]	89.7 ± 10.7	85.2 ± 7.4	1.8378, 0.6431
Regular vitamin D supplements user [*n* (%)]	92 (45.8)	112 (86.2)	2.5032, <0.0001
Physical activity			
Insufficient	45 (22.4)	10 (7.7)	1.3432, 0.0034
Sufficient	114 (56.7)	61 (46.9)	0.9468, 0.0051
Increased	31 (15.4)	33 (25.4)	0.9456, 0.0013
High	11 (5.5)	26 (20.0)	1.6874, 0.0041
Dietary intake			
Energy [kcal/day]	1523.7 ± 587.1	2171.8 ± 782.7	1.9563, <0.0001
Proteins [g/day]	56.8 ± 15.8	87.6 ± 51.1	1.9037, <0.0001
Proteins [% total energy intake]	17.1 ± 6.9	22.4 ± 3.7	2.0962, <0.0001
Carbohydrates [g/day]	197.9 ± 56.5	231.8 ± 178.1	2.7094, <0.0001
Carbohydrates [% total energy intake]	54.1 ± 7.2	50.7 ± 6.5	0.9373, 0.0023
Fats [g/day]	58.8 ± 23.4	80.7 ± 47.4	1.9404, <0.0001
Fats [% total energy intake]	32.5 ± 8.7	33.6 ± 7.1	0.4592, 0.4953
MUFA [g/day]	19.3 ± 7.8	31.1 ± 19.7	3.3982, <0.0001
PUFA [g/day]	6.9 ± 5.6	14.1 ± 3.5	2.9934, <0.0001
SFA [g/day]	27.2 ± 7.3	35.4 ± 18.9	2.7073, <0.0001
Fiber [g/day]	11.9 ± 4.2	28.3 ± 8.7	4.2028, <0.0001
Vitamin D (without supplements) [µg/day]	3.4 ± 2.7	8.9 ± 5.1	1.4541, <0.0001
Total vitamin D (including supplements) [µg/day]	34.5 ± 11.9	26.3 ± 11.8	2.6734, 0.0076
Serum concentration			
25(OH)D Total [ng/mL]	20.5 ± 3.1	28.2 ± 3.8	5.0276, <0.0001
25(OH)D deficiency [*n* (%)]	151 (75.1)	66 (50.8)	1.2736, <0.0001

## Data Availability

Publicly available datasets were not analyzed in this study.
